# Do follicles matter? Testing the effect of follicles on hair cortisol levels

**DOI:** 10.1093/conphys/coaa003

**Published:** 2020-02-01

**Authors:** Agnieszka Sergiel, Marc Cattet, Luciene Kapronczai, David M Janz, Nuria Selva, Kamil A Bartoń, Jon E Swenson, Andreas Zedrosser

**Affiliations:** 1 Institute of Nature Conservation, Polish Academy of Sciences, Adama Mickiewicza 33, 31120 Krakow, Poland; 2 RGL Recovery Wildlife Health & Veterinary Services, 415 Mount Allison Crescent, Saskatoon, Saskatchewan, S7H 4A6, Canada; 3 Department of Veterinary Pathology, University of Saskatchewan, 52 Campus Drive, Saskatoon, Saskatchewan, S7N 5B4, Canada; 4 Toxicology Centre, University of Saskatchewan, 44 Campus Drive, Saskatoon, Saskatchewan, S7N 5B3, Canada; 5 Department of Veterinary Biomedical Sciences, University of Saskatchewan, 52 Campus Drive, Saskatoon, Saskatchewan, S7N 5B4, Canada; 6 Faculty of Environmental Science and Natural Resource Management, Norwegian University of Life Sciences, Høgskoleveien 12, NO-1432 Ås, Norway; 7 Department of Natural Sciences and Environmental Health, University of South-Eastern Norway, Gullbringvegen 36, 3800 Bø, Norway; 8 Institute for Wildlife Biology and Game Management, University for Natural Resources and Life Sciences, Gregor-Mendel-Straße 33, 1180 Vienna, Austria

**Keywords:** Hair cortisol extraction methods, hair sampling, physiological ecology, stress

## Abstract

Cortisol concentrations in hair are used increasingly as a biomarker of long-term stress in free-ranging wildlife. Cortisol is believed to be integrated into hair primarily during its active growth phase, typically occurring over weeks to months or longer periods, depending on latitude. Cortisol concentrations in hair thus reflect the activity of the hypothalamic–pituitary–adrenal axis over this time. However, local, independent cortisol secretion within the skin, which includes hair follicles, may also contribute to cortisol levels in growing hair. Methodological differences between studies include the measurement of cortisol in only the hair shaft (i.e. follicle absent, as with shaved hair) versus the whole hair (i.e. follicle present, as with plucked hair). If the concentration of cortisol in the follicle is high enough to influence the overall hair cortisol concentration (HCC), this could confound comparisons between studies using different types of hair samples (hair shafts vs. whole hair) and collection methods. Here, we test the hypothesis that cortisol present in follicles influences HCC. We compared HCC in paired subsamples of hair with and without follicles from 30 free-ranging Scandinavian brown bears (*Ursus arctos*) and observed significantly greater HCC in samples with follicles present. The effect of follicles remained significant also with sex and age of sampled bears taken into account in a linear mixed model. Finally, we provide an overview of collection methods and types of hair samples used for HCC analysis in 77 studies dealing with stress in wild mammal species. Our findings highlight the need to unify methods of hair collection and preparation to allow for valid comparisons, and to optimize labour input in ecophysiological studies.

## Teaser

To determine the potential influence of hair follicles on cortisol levels, we conducted an experiment comparing cortisol concentrations in paired subsamples of brown bear hair with and without follicles. We found significantly greater cortisol values in subsamples that included follicles, suggesting that the presence of follicles may influence cortisol concentrations measured in hair. Our findings underscore the importance of standardizing hair collection and preparation methods to enable comparisons across studies.

## Introduction

Hair is a source of biological information for genetics (Chang *et al.*, 2002; Belant *et al.*, 2007; Kendall *et al.*, 2009; Henry *et al*., 2011), dietary analysis (McLaren *et al.*, 2015; Kaczensky *et al.*, 2017), environmental exposure (McLean *et al.*, 2009; Shah *et al.*, 2011; Gillespie *et al.*, 2012; Bechshøft *et al.*, 2015; Carlitz *et al.*, 2016; Waterhouse *et al.*, 2017), health (Crowley *et al.*, 2018) and reproductive status (Cattet *et al.*, 2017; Cattet *et al.*, 2018). Concentrations of hormones (Macbeth *et al.*, 2010), pollutants (Bechshøft *et al.*, 2012, 2015) or toxins (Kintz, 2004) incorporated into hair can be measured years to centuries after their deposition (Kintz, 2004; Webb *et al.*, 2010). Compared to blood, saliva, urine or faeces, hair is a relatively stable medium that is easy to collect and can be transported and stored at room temperature (Felicetti *et al.*, 2003, Jaspers *et al.*, 2010). Therefore, in the last two decades, hair has become a recognized medium to study long-term stress in wildlife via the measurement of cortisol within the hair shaft (Koren *et al.*, 2002; Macbeth *et al*., 2010; Sheriff *et al.*, 2011; Macbeth *et al.*, 2012; Cattet *et al.*, 2014; Mastromonaco *et al.*, 2014; Rakotoniaina *et al.*, 2017).

Hair growth follows a cyclic pattern of active growth alternated with quiescence (Pilkus and Chuong, 2008; Müntener *et al.*, 2011) but is not yet well characterized for most mammals. Certain drugs, toxins, metabolites and hormones circulating in the blood are incorporated into the hair shaft medulla primarily during anagen, i.e. the active growth phase (Harkey, 1993; Davenport *et al.*, 2006; Pragst and Balikova, 2006). Although the duration of anagen is species-specific, it typically lasts weeks to months (e.g. Samuel *et al.*, 1986; Cuyler and Ørtisland, 2002). Substances may also enter the root or shaft of the growing hair (with some temporal delay) via diffusion from the external environment or tissues surrounding actively growing hair, or as the result of glandular apocrine, sebaceous and sweat secretions in and around the follicle (Henderson, 1993; Pragst and Balikova, 2006). Moreover, a parallel corticotropin-releasing hormone (CRH), adrenocorticotropic hormone and cortisol production system has been demonstrated within the skin, including its epidermal and dermal compartments, as well as hair follicular cells (Bamberg *et al.*, 2005; Sharpley *et al.*, 2011; Keckeis *et al.*, 2012; Jozic *et al.*, 2014). Ito *et al.* (2005) reported that human scalp hair follicles grown *in vitro* are capable of responding directly to CRH stimulation, including cortisol production and the activation of regulatory feedback loops. The intrafollicular cortisol production occurred long after its disconnection from the systemic hypothalamic–pituitary–adrenal (HPA) axis and any neural, vascular or extrafollicular stimuli and was maintained under *in vitro* conditions.

Brown bears (*Ursus arctos*) exhibit a single annual moulting period, with seasonal hair follicle activity occurring from spring to fall (Felicetti *et al.*, 2003; Jones *et al.*, 2006; Christensen *et al.*, 2007). During this time, the majority of hair follicles grow actively and incorporate cortisol and other substances (Stenn and Paus, 2001; Davenport *et al.*, 2006; Pragst and Balikova, 2006). Hair growth in brown bears has been estimated to occur at a rate of ~500 μm per day, and thus each 1 cm of hair may reflect a period of exposure of about 20 days (Christensen *et al.*, 2007). Therefore, cortisol concentrations in hair should represent HPA axis activity occurring during this time period (Macbeth *et al.*, 2010; Sheriff *et al.*, 2011). Differences in hair cortisol concentration (HCC) between sex and/or reproductive classes have been reported (e.g. Macbeth *et al.*, 2012). A seasonal pattern, with greater HCC in spring than fall, was also reported in brown bears (Cattet *et al.*, 2018). Additionally, the difference in cortisol levels between age classes was more pronounced during spring.

Plucking and snagging hair, which mostly includes the collection of hair follicles, are routine methods to collect hair samples from wildlife, for example to extract DNA from the follicles (e.g. Kendall *et al.*, 2009). In addition, hair samples are often collected by shaving or cutting directly from the animal body, i.e. hair follicles are not collected (e.g. Macbeth *et al.*, 2010; Brearley *et al*., 2012; Mastromonaco *et al.*, 2014). Samples collected using any of these methods can be used for cortisol analysis. Several studies have used full hair strands (i.e. follicles included) for cortisol determination (Koren *et al*., 2002; Caslini *et al*., 2016). However, Macbeth *et al.* (2010) and Cattet *et al.* (2014) have suggested to remove hair follicles for cortisol analyses to avoid potential differences in hormone concentration due to the presence of follicles (Cattet *et al.,* 2017), while the manual removal of follicles from plucked or snagged hair samples is highly time-consuming. If the presence or absence of hair follicles does not significantly affect the HCC, the efficiency of hair cortisol extraction could be improved markedly, as all methods of collection, i.e. snagging, plucking and shaving, would be equally suitable for long-term stress studies. Therefore, it is important to address the presence vs. absence of hair follicles and their potential influence on results in hormone analyses (i) to better understand the mechanisms of hair cortisol deposition and (ii) to contribute to the standardization of hair cortisol quantification methods.

Here, we determined cortisol concentrations in hair of wild brown bears collected on the same day from the same body region, but extracted using two methods (including and excluding follicles), to test the hypothesis that HCC is influenced by the presence/absence of follicles. We also investigated the potential influence of age and sex of bears on HCC determined in hair with follicles present or absent. In addition, we present an overview of studies determining HCC in wild mammals, with a focus on the hair collection method and sample type (i.e. presence or absence of follicles).

## Material and methods

### Sample selection and collection method

We randomly selected 30 guard hair samples from 30 free-ranging brown bears within a large pool of samples collected by the Scandinavian Brown Bear Research Project. The basic and only criteria were that the samples were of sufficient quantity for subdivision into two subsamples and free of visible external debris (e.g. blood, dirt). All sampled bears were captured by remote drug delivery from a helicopter within two weeks after leaving the den in the spring of 2002–2006, following procedures as described in Arnemo and Evans (2017) and approved by the appropriate authorities (Swedish Environmental Protection Agency, Stockholm: #NF-412-4762; Swedish Board of Agriculture: #35-846; Swedish Ethical Committee on Animal Research, Uppsala: #277, #40 and #C59). All samples were collected by plucking the guard hairs with pliers from a standardized location between the shoulder blades. Samples were air-dried and had been stored in the dark at room temperature since transportation from the field.

### Sample preparation and hair cortisol analysis

Each of the 30 hair samples was subdivided into two subsamples of ≥50 mg, with the subsamples being as identical as possible in relation to visual appearance. In Subsample 1, follicles were removed with scissors; in Subsample 2, hair was left with follicles intact. This approach allowed a pairwise comparison of Subsamples 1 and 2, collected at the same time and from the same site from the same individuals. Processing and extracting procedures, as well as cortisol analysis, were conducted following the protocol described by Macbeth *et al.* (2010). HCCs were determined using a commercially available enzyme-linked immunosorbent assay (ELISA) kit (Oxford Biomedical, Rochester Hills, MI, USA) and standardized to hair mass finally used for extraction (≥25 mg). Cross-reactivity values of this assay for cortisol, cortisone, 11-deoxycortisol, corticosterone, 6-β-hydroxycortisol, 17-hydroxyprogesterone and deoxycorticosterone are 100, 15.77, 15, 4.81, 1.37, 1.36 and 0.94%, respectively. Extracts were run in duplicates on the ELISA, with intra- and inter-assay percent coefficients of variation (% CV; SD/mean × 100%) of 4.9 and 5.1%, respectively.

### Statistical analysis of HCC

We initially performed a Shapiro–Wilk normality test and used a paired *t* test to determine if mean HCC was affected by the presence or absence of hair follicles. To test whether the potential difference was affected by sex and/or age of individuals, we fitted a linear mixed model (LMM) including these variables as fixed effects, at the same time accounting for the inter-assay and between-individual variation in the effect of sample type. The model included individual age (log-transformed to normalize the variance), sex and sample type with all their two-way interactions as fixed effects (the three-way interaction was removed as it was non-significant), with random effects modelled with sample type (random ‘slope’) and grouped by the sample (individual) and plate. Therefore, the effect of sample type was simultaneously modelled both as random and fixed. Finally, to test whether there was indeed a systematic difference in HCC associated with sample type, we compared this model with the one excluding the fixed effect of the sample type, using a likelihood ratio test (LRT). The analyses were performed in R 3.6.0 (R Core Team, 2019) with the package ‘nlme’ (Pinheiro *et al.*, 2018) and ‘car’ (for ‘Anova’).

### Literature search

To evaluate the methods commonly used for hair collection and types of hair samples used for cortisol analysis, we thoroughly searched the Google Scholar database from its inception until the end of 2018, using the keywords ‘hair’ and ‘cortisol’ combined into a single search string, sorted by relevance, and only including wild mammalian species. This search yielded a total of 4090 papers. We excluded laboratory rodents (for which corticosterone is the predominant glucocorticoid; Gong *et al*., 2015), domestic animals and humans, in order to cover only the methods used in wildlife ecophysiological studies. References were then selected for inclusion into our review according to the following criteria: (i) the study must include the measurement of HCCs in a wild mammal species; (ii) must be published as a peer-reviewed article; and (iii) must be published in English. The papers were screened for hair sample collection details and whether extractions were conducted with follicles absent or present in the sample.

## Results

### HCC

**Figure 1:**
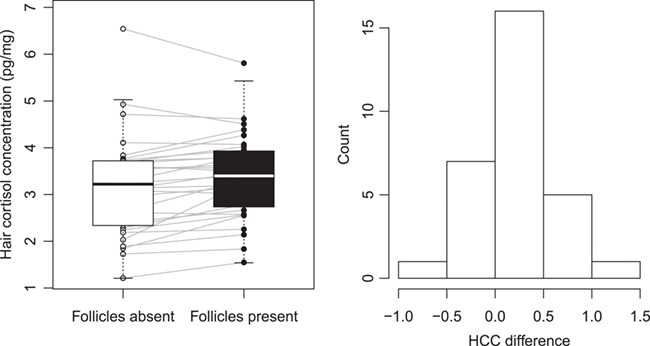
Hair cortisol concentrations (HCC) in paired subsamples of 30 brown bears, collected in Sweden from 2002 to 2006, extracted with hair follicles removed compared to subsamples extracted with hair follicles intact (left), with histogram of pairwise HCC difference (follicles present–absent; right)

Our selection procedure resulted in a sample set comprised of three age classes: yearling (eight individuals—four males and four females), subadult (2–4 years old, 10 individuals—five males and five females) and adult bears (5–24 years old, 12 individuals—six males and six females). Comparing all samples (*n* = 30), we found significantly greater HCC in paired subsamples containing follicles (mean = 3.39 ± 0.916 (SD) pg/mg) compared to subsamples without follicles (mean = 3.18 ± 1.092 (SD) pg/mg; paired sample *t* test, *t* = −3.173, *P* = 0.004; Fig. 1). Pairwise HCC difference (follicles present–absent) ranged from −0.73 to 1.20 (Fig. 1). In 22 (73%) of samples, HCC was higher in the subsample with follicles. Mean difference between two sample types was 0.206 ± 0.07 SD pg/mg (6.6% higher on average in subsamples with follicles).

**Figure 2:**
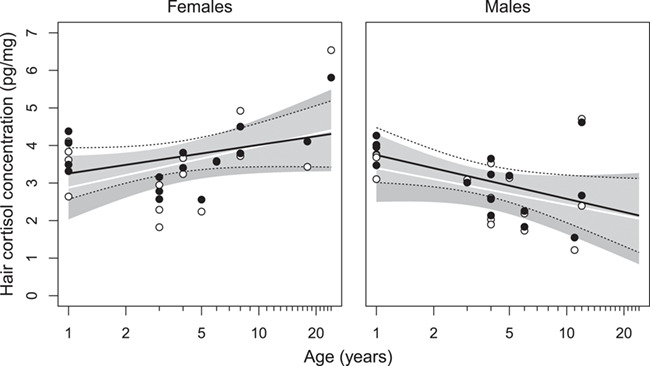
Hair cortisol concentration in samples extracted with follicles present (filled bullets) and absent (white bullets), broken down by sex, against individual age (on log-scaled *x*-axis). Lines show predictions of the linear mixed effect model (see Methods)—mean ± 95% confidence intervals (black solid and dotted lines for follicles present, and white line and gray shading for follicles absent)

The extended model including sex and age effects showed that HCC increased with age in females, but decreased in males, with a significant between-sex difference between the directions of the relationship (Fig. 2; see also Supplementary Tables 1 and 2). Additionally, this model indicated that while the sample type affected HCC, this effect tended to diminish with individual age (indicated by a negative, nearly significant interaction of ‘Sample type’ and ‘Age’; Supplementary Table 2 and Fig. 2). Nevertheless, the overall effect of the sample type was highly significant (LRT, *χ*^2^ = 12.7, *P* = 0.005).

### Review of literature

We selected 77 papers published between January 2002 and December 2018 (Supplementary Table 3). The majority of the reviewed studies (64/77–83%) reported analytical validations of the methods used, while biological validation was the only validation reported in 13 studies. Physiological validation by ACTH (adrenocorticotropic hormone) challenge has been reported for three species. Most (55.8%) of the reviewed studies used samples from captive animals (43/77, mostly primates—over 80% of studies on primates were conducted on captive animals) and 32 (41.6%) used samples collected from free-ranging animals. In two studies, both captive and free-ranging individuals were used. The summary and list of references used in the review are included as Supplementary material (Supplementary Table 3).

**Figure 3:**
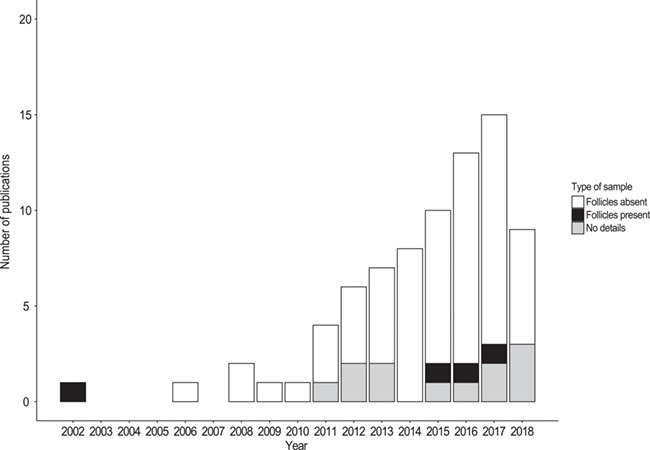
The number of peer-reviewed studies reporting hair cortisol analysis in wild animal species from January 2002 to December 2018, broken down by type of hair sample used for cortisol extraction (follicles absent, present or lacking those details)

The first paper was published in 2002, where HCC was determined in free-ranging rock hyrax (*Procavia capensis*) (Koren *et al.,* 2002), followed by only four papers published during 2006–2009 on captive primates (Davenport *et al.*, 2006; Clara *et al.*, 2008; Davenport *et al.*, 2008; Dettmer *et al.*, 2009). Since 2010, the number of papers reporting HCC in wild animal species has increased steadily (Fig. 3). In this year, Macbeth *et al.* (2010) published a study on free-ranging brown bears and promoted a wider application of hair for wildlife stress research. The authors used hair collected by three methods (shaving, plucking and snagging), but all samples used for extraction were unified by manual removal of the follicles.

Manual removal of the follicles before processing and extraction was included in seven studies (9.1%), whereas follicles were included in four studies (Fig. 3, Supplementary Table 3). Twelve papers (15.6%) contained no details on the type of sample used for extraction. In 37 studies (48.1%), hair samples were collected by shaving; therefore, only hair shafts (no follicles) were processed and extracted. The second most common method of hair collection was clipping as close to the skin as possible (11 studies, 14.3%), and only in a few studies were plucked, snagged, stripped or naturally shed hair samples used (one, two, one and one study, respectively; Supplementary Table 3). We found nine studies that used several methods of hair collection, of which only one study used a comparative approach, i.e. using samples collected by plucking so the presence of follicles could be taken into account as a variable (Cattet *et al.*, 2017). No information on hair collection method was provided in 14 studies (18.2%).

## Discussion

We confirmed our hypothesis that HCC is influenced by the presence/absence of follicles in the extracted sample. Our results showed that samples extracted with follicles present had on average 6.6% (0.21 pg/mg) greater HCC values compared to samples with follicles removed. Variation among samples within an ELISA plate (intra-assay %CV) and between ELISA plates analyzed on separate days (inter-assay %CV) was 4.9 and 5.1%, respectively, suggesting high precision achieved in the technique used to quantify HCC, and so we are confident that the mean difference in values between paired samples with or without follicles higher than the variation in precision, warrants consideration when using hair as a medium. Although the difference in our experiment does not appear considerable, comparing to other sources of variations, e.g. ~ 1.3–1.8 pg/mg greater HCC between the neck and other body regions in brown bears (Macbeth *et al.*, 2010), it was higher than intra- and inter-assay CV, statistically significant, and the presence of follicles remained as a significant factor when sex and age were considered. In comparison, HCC in paired hair samples with and without follicles reported in Cattet *et al*. (2017) and analysed with the same commercial assay kit, but in captive bears, were also greater for the subsamples extracted with follicles, although not statistically significant (mean with follicles—1.16 pg/mg vs. mean without follicles—1.06 pg/mg, *n* = 20; paired t-test result—*P* = 0.082). Additionally, hair progesterone concentrations were on average almost twice as high in samples extracted with follicles (7.03 pg/mg) vs. samples without follicles (3.91 pg/mg; Cattet *et al*., 2017).

The follicle is a dynamic miniorgan, highly sensitive to external (environmental) stimuli and numerous endogenous factors, such as hormones and cytokines, which in part are produced by the follicle itself (Schneider *et al.*, 2009). Apart from this sensitivity, its cycling is so autonomous that the follicle is able to continue activity even while isolated in culture (Ito *et al.*, 2005; Schneider *et al.*, 2009). It has been shown that isolated human hair follicles secrete substantial levels of cortisol into the culture medium, and this activity is further up-regulated by CRH. CRH also modulates important functional hair growth parameters *in vitro* (hair shaft elongation, catagen induction, hair keratinocyte proliferation, melanin production). Finally, as shown for human hair, follicles display HPA axis-like regulatory feedback systems, because the glucocorticoid receptor agonist, hydrocortisone, down-regulates follicular CRH expression. Thus, even in the absence of endocrine, neural or vascular systemic connections, hair follicles directly respond to CRH stimulation in a strikingly similar manner to what is seen in the classical HPA axis, including synthesis and secretion of cortisol and activation of prototypic neuroendocrine feedback loops (Ito *et al.*, 2005). It is unknown how long the follicle or its removed parts while collecting hair, e.g. by plucking, can remain active and/or alive when disconnected from the skin. Additionally, it is also possible that cortisol levels in hairs with follicles are higher due to residual blood remaining in the follicle after the wash process.

The use of HCC as a biomarker of long-term stress has several advantages. By knowing the hair growth rate (e.g. ~1 cm per 20 days in brown bears; Christensen *et al.*, 2007), a retrospective examination of cortisol deposition can be determined. Alternatively, HCC can provide a baseline cortisol assessment for a time period during which the effect of a stressor had not yet occurred, as demonstrated in rhesus macaques (*Macaca mulatta*) sampled both at baseline and after a major stressful event (relocation to a new enclosure; Davenport *et al*., 2006). In addition, and unlike other matrices (e.g. blood, urine, saliva) that require special storage conditions prior to analysis, hair samples are easily transported and stored in paper envelopes in the dark at room temperature for extended time periods. Another advantage is that the stability of hair cortisol allows the use of archaeological and historical samples, as shown in studies comparing cortisol levels in hair of modern humans with that of excavated human remains (e.g. Webb *et al.*, 2010).

Brown bears moult once annually, and active hair growth is believed to stop at the onset of hibernation (Christensen *et al.*, 2007) and to resume again after emergence from hibernation in spring (Schwartz *et al.*, 2003). Additionally, it has been suggested that latitude, sex and age may affect hair growth, and its onset and duration may be highly variable in individual bears (Pearson, 1975; Schwartz *et al.*, 2003). The activity of hair follicles is intermittent, consisting of active (anagen), transitional (catagen) and resting (telogen) phases. Thus, HCC may vary in relation to the hair growth rate (Felicetti *et al.*, 2003; Jones *et al.*, 2006; Christensen *et al.*, 2007; Sauvé *et al.*, 2007), and therefore season and date of collection may have an important effect on HCC, as shown by Cattet *et al.* (2017). Studies show also an effect of sex and/or reproductive status, as well as age, on HCC and its inter-individual variation (Macbeth *et al.*, 2012; Cattet *et al*., 2018). Apart of those intrinsic sources of differences in HCC, previous validation studies also confirmed an effect of e.g. body region (Macbeth *et al.*, 2010; Fourie *et al.*, 2016), sample mass (Fourie *et al.*, 2016) or incubation technique (Fourie *et al.*, 2016). As the steroids sequestered in hair are derived from various sources, including skin (Bamberg *et al.*, 2005; Ito *et al.*, 2005; Keckeis *et al.*, 2012), it is important to note that hormones in hair may reflect a combination of long- and short-term levels with an unknown relative contribution of different sources (Koren *et al.*, 2019). Therefore, whether the magnitude of difference as shown in our study is biologically meaningful remains debatable. As mentioned above while referring to the validation studies, HCC may vary in individual animals depending on where the hair is collected from on the body, and certain body regions show consistent differences when compared with others (e.g. hair from the neck and the head showed consistently higher HCC in the study by Macbeth *et al.*, 2010). In contrast, in the study on orangutans (*Pongo* spp.) by Carlitz *et al*. (2014) no significant differences between defined body regions (right wrist, left wrist, stomach, back, left shoulder and right shoulder) were found, with percentage difference from the mean HCC oscillating around 5%. Given that samples from captured bears in this study were collected by plucking from a standardized location between the shoulder blades and divided into subsamples before the experiment, it is unlikely that some systematic sampling bias could fully explain the differences in HCC values between pairs of subsamples. When considering the studies testing HCC as a marker for no stress vs. stress situations, as in the study by Fairbanks *et al*. (2011) on vervet monkeys (*Chlorocebus aethiops sabaeus*) in baseline and post-move sampling, the difference between non-stressful and stressful conditions for individuals appeared as a 27% mean increase in HCC, while 15% of individuals had equal or even lower values post-move. Cattet *et al.* (2014) reported that HCC in brown bears sampled following capture by three methods (helicopter, Aldrich snare or culvert trap) were more likely to have a greater HCC (median 2.30 pg/mg) than free-ranging bears sampled non-invasively by barbed wire (0.94 pg/mg). Obviously, the magnitude of difference varies among studies with different research questions and objectives, and the level of analyses. As shown above on the example of vervet monkeys and also in our study, there are instances where the individual results appear counter-intuitive. Nevertheless, the possible sources of variation, especially ones that can be assigned to sample collection, processing and extraction require systematic testing to be minimized and allow any comparisons across studies.

The analysis of HCC involves a number of steps prior to the immunoassay, such as collecting samples from certain body region using various methods, storage, weighing, washing, grinding the samples, extracting and drying the extract and finally reconstituting in a buffer for analysis (see e.g. Macbeth *et al.*, 2010). Some of these factors have not been systematically assessed for their effect on HCC, although processing and subsequent extraction methods and their effect on HCC (see e.g. Kroshko *et al.*, 2017; Sergiel *et al.*, 2017) are important for comparisons across studies. Similarly, exposure to ultraviolet radiation (UV) and mechanical treatment (e.g. extensive brushing) have been recently reported to influence HCC (Salaberger *et al.*, 2016; Wester *et al.*, 2016). In some of the published studies, there was no explicit information about whether the hair samples were extracted with or without follicles, and all those refer back to papers with details about the methods used (e.g. Bechshøft *et al*., 2012 referring to paper by Davenport *et al*., 2006, where the collected hair samples were shaved and therefore contained no follicles). In stress studies, shaving appears to be the most common method (e.g. Davenport *et al*., 2006; Corradini *et al*., 2013), but also plucking and snagging is used (Bourbonnais *et al.*, 2013; Cattet *et al*., 2014; Sergiel *et al*., 2017), and the latter requires manual removal of follicles (e.g. Macbeth *et al.*, 2010; Cattet *et al.*, 2014). Still, there are papers where the details on collection and sample type used for extraction are neglected. The question of a potential follicle effect on HCC remains important, as hair is used for many types of ecological (e.g. Bechshøft *et al.*, 2012), physiological (e.g. Cattet *et al.*, 2014) and genetic (e.g. Ausband *et al.*, 2011) studies, and hair samples initially collected for genetics can be used further to answer ecophysiological questions, after purpose- and analysis-specific processing (Bryan *et al.*, 2013; Cattet *et al*., 2014).

As shown in this study, there was an influence of follicles on hair hormone concentrations, and despite the difference being significant or negligible in comparison to other sources of variation, this effect is to be considered. We believe that for the purpose of standardization in wildlife studies using hair hormone concentrations, whether follicles were included into extracted samples or not is methodologically crucial and should be reported.

## Conclusions & recommendations

A diversity of sample collection and processing methods has been employed in studies involving HCC analyses in wild mammals. We propose that standardized guidelines for sample collection and extraction should be adopted, because the presence of follicles may influence HCC, possibly resulting from residual blood remaining in follicles, from cortisol production within the follicle or from a combination of both factors. Statistical analyses, and especially comparisons across populations, species and studies, must take the absence or presence of follicles into account when working with HCC. Similarly, preliminary results using other steroid hormones suggest that this should be considered for other hormonal analyses using hair as a medium.

## Supplementary Material

Sergiel_et-al_Do_follicles_matter_Supplementary_material_3_coaa003
