# Supplementary material for: Do follicles matter? Testing the effect of follicles on hair cortisol levels
Source: Conserv Physiol. 2020 Feb 1;8(1):coaa003. doi: 10.1093/conphys/coaa003 (PMC6994724; doi:10.1093/conphys/coaa003)
Supplement: Sergiel_et-al_Do_follicles_matter_Supplementary_material_3_coaa003 [file sergiel_et-al_do_follicles_matter_supplementary_material_3_coaa003.pdf]

Supplementary material to:

Do follicles matter? Testing the effect of follicles on hair cortisol levels

Agnieszka Sergiel, Marc Cattet, Luciene Kapronczai, David M. Janz, Nuria Selva, Kamil A. Bartoń, Jon E. Swenson and Andreas Zedrosser

Supplementary Table 1: Comparison of hair cortisol concentration (pg/mg) measured in paired subsamples, and sex and age data of 30 Scandinavian brown bear individuals.

Sample ID	HCC (pg/mg) with follicles absent	HCC (pg/mg) with follicles present	Sex	Age
1	2.64	3.32	Female	1
2	3.62	3.49	Female	1
3	3.84	4.38	Female	1
4	4.11	4.07	Female	1
5	1.82	2.57	Female	3
6	2.29	2.78	Female	3
7	2.95	3.16	Female	3
8	3.24	3.41	Female	4
9	3.66	3.81	Female	4
10	2.24	2.56	Female	5
11	3.59	3.57	Female	6
12	3.72	3.79	Female	8
13	4.92	4.50	Female	8

14	3.43	4.11	Female	18
15	6.54	5.81	Female	24
**Mean ± SD (Females)**	**3.51±1.160**	**3.69±0.840**		
16	3.10	3.47	Male	1
17	3.68	4.02	Male	1
18	3.74	3.96	Male	1
19	3.76	4.27	Male	1
20	3.09	3.01	Male	3
21	1.90	2.14	Male	4
22	2.03	3.23	Male	4
23	2.61	2.57	Male	4
24	3.52	3.65	Male	4
25	3.14	3.20	Male	5
26	1.73	1.84	Male	6
27	2.19	2.26	Male	6
28	1.22	1.55	Male	11
29	2.39	2.67	Male	12
30	4.71	4.62	Male	12
**Mean ± SD (Males)**	**2.85±0.949**	**3.10±0.919**		
**Mean ± SD (Both)**	**3.18±1.093**	**3.39±0.916**		

Supplementary Table 2: Results of the linear mixed effect model explaining hair cortisol concentration with an interaction of individual sex, age, and sample type (type III ANOVA). The model included random effects of plate and sample.

	df	F-value	p-value
**(Intercept)**	87	138,9	< 0.0001
**Sample type**	87	11,6	0.001
**Sex**	24	0,5	0.5
**log(Age)**	24	0,0	0.8
**Sample type : Sex**	87	0,3	0.6
**Sample type * log(Age)**	87	3,5	0.06
**Sex * log(Age)**	24	6,1	0.02

Supplementary Table 3: Summary of the literature review on the methods of collection and type of hair samples (with or without follicles) used for cortisol extraction in wild mammal species, both free-ranging and captive.

Species	Status of studied animals	Hair collection method	Type of hair samples used for cortisol extraction (follicles absent or present)	Reference
Rock hyrax (*Procavia capensis*)	Free-ranging	Plucking	Follicles present	Koren *et al*., 2002
Rhesus macaque (*Macaca mulatta*)	Captive	Shaving	Follicles absent	Davenport *et al*., 2006
Common marmoset (*Callithrix jacchus*)	Captive	Cutting	Follicles absent	Clara *et al*., 2008
Rhesus macaque (*Macaca mulatta*)	Captive	Shaving	Follicles absent	Davenport *et al*., 2008
Rhesus macaque (*Macaca mulatta*)	Captive	Shaving	Follicles absent	Dettmer *et al*., 2009
Brown bear (*Ursus arctos*)	Free-ranging	Shaving<br>Plucking<br>Snagging	Follicles absent<br>Follicles present but manually removed before processing<br>Follicles present but manually removed before processing	Macbeth *et al*., 2010
Caribou (*Rangifer tarandus*	Captive	Shaving	Follicles absent	Ashley *et al*., 2011

granti)				
Reindeer (*Rangifer tarandus tarandus*)	Captive			
Polar bear (*Ursus maritimus*)	Free-ranging	No details	No details; hair samples were processed for cortisol analysis following method described in Davenport *et al.* 2006	Bechshøft *et al*., 2011
Vervet monkey (*Chlorocebus aethiops sabaeus*)	Captive	Shaving	Follicles absent	Fairbanks *et al*., 2011
Ring-tailed lemur (*Lemur catta*)	Captive	Shaving	Follicles absent	Fourie *et al*., 2011
Coquerel's sifaka (*Propithecus verreauxi coquereli*)	Captive			
Grivet monkey (*Chlorocebus aethiops*)	Captive			
Golden-bellied mangabey (*Cercocebus chrysogaster*)	Free-ranging			
Patas monkey (*Erythrocebus patas*)	Captive			
Hamadryas baboon (*Papio hamadryas papio*)	Captive			
Chacma baboon (*Papio hamadryas ursinus*)	Free ranging			
Western lowland gorilla	Captive			

(*Gorilla gorilla gorilla*)				
Nancy Ma's night monkey (*Aotus nancymaae*)	Captive			
Common marmoset (*Callithrix jacchus*)	Captive			
Tufted capuchin (*Cebus apella*)	Captive			
White-faced saki (*Pithecia pithecia*)	Captive			
Cotton-headed tamarin (*Saguinus oedipus*)	Captive			
Polar bear (*Ursus maritimus*)	Free-ranging	No details	No details; cortisol analysis referenced to Bechshøft *et al.* 2011 (where the methods are lacking sampling details but are referenced to Davenport *et al.* 2006)	Bechshøft, Rigét *et al*., 2012
Polar bear (*Ursus maritimus*)	Free-ranging	No details	No details; reference to Bechshøft *et al.* 2011 (where the methods are lacking sampling details but are referenced to Davenport *et al.* 2006)	Bechshøft, Sonne *et al*., 2012
Squirrel glider (*Petaurus norfolcensis*)	Free-ranging	Shaving	Follicles absent	Brearley *et al*., 2012
Rhesus macaque (*Macaca mulatta*)	Captive	Shaving	Follicles absent	Dettmer *et al*., 2012

Polar bear (*Ursus maritimus*)	Free-ranging	No details	Hair shafts (no follicles) used for extraction; reference to Macbeth *et al.* 2010	Macbeth *et al*., 2012
Polar bear (*Ursus maritimus*)	Free-ranging	No details	No details; reference to Bechshøft *et al.* 2011 (where the methods are also lacking sampling details but are referenced to Davenport *et al.* 2006)	Bechshøft *et al*., 2013
Brown bear (*Ursus arctos*)	Free-ranging	Snagging<br>Cutting/shaving	Follicles removed for genetics and only hair shaft used for hormone assay<br>Follicles absent	Bryan *et al*., 2013
Brown bear (*Ursus arctos*)	Free-ranging	Snagging	No details, referenced to Macbeth *et al.* 2010	Bourbonnais *et al*., 2013
Bonnet macaque (*Macaca radiata*)	Captive	No details	No details, referenced to Davenport *et al.* 2006	Laudenslager *et al*., 2013
Asiatic black bear (*Ursus thibetanus*)	Captive	Shaving	Follicles absent	Malcolm *et al*., 2013
Rhesus macaque (*Macaca mulatta*)	Captive	Cutting	Follicles absent	Qin *et al*., 2013
Canada lynx (*Lynx canadensis*)	Captive	Cutting	Follicles absent	Terwissen *et al*., 2013

Chimpanzee (*Pan troglodytes*)	Captive	Cutting	Follicles absent	Yamanashi *et al.*, 2013
Rhesus macaque (*Macaca mulatta*)	Captive	Shaving	Follicles absent	Dettmer *et al.*, 2014
Orangutan (*Pongo* spp.)	Captive	Shaving	Follicles absent	Carlitz *et al.*, 2014
Brown bear (*Ursus arctos*)	Free-ranging	Plucking	Follicles present but manually removed before processing	Cattet *et al.*, 2014
		Snagging	Follicles present but manually removed before processing	
Crab-eating macaque (*Macaca fascicularis*)	Captive	Cutting	Follicles absent	Chu *et al.*, 2014
Rhesus macaque (*Macaca mulatta*)	Captive	Shaving	Follicles absent	Kapoor *et al.*, 2014
Eastern chipmunk (*Tamias striatus*)	Free-ranging	Shaving	Follicles absent	Mastromonaco *et al.*, 2014
Rhesus macaque (*Macaca mulatta*)	Captive	Shaving	Follicles absent	Meyer *et al.*, 2014
Rhesus macaque (*Macaca mulatta*)	Captive	Shaving	Follicles absent	Novak *et al.*, 2014
Polar bears (*Ursus maritimus*)	Free-ranging	No details	No details, reference to Macbeth *et al.* 2010	Bechshøft *et al.*, 2015
Grey wolf (*Canis lupus*)	Free-ranging	Cutting	Follicles absent	Bryan *et al.*, 2015

Chimpanzee (*Pan troglodytes*)	Captive	Cutting	Follicles absent	Carlitz *et al*., 2015
Rhesus macaque (*Macaca mulatta*)	Captive	Shaving	Follicles absent	Dettmer, Rosenberg, Menard *et al*., 2015
Rhesus macaque (*Macaca mulatta*)	Captive	Shaving	Follicles absent	Dettmer, Rosenberg, Suomi *et al*., 2015
Olive baboon (*Papio anubis*)<br>Hamadryas baboon (*Papio hamadryas*)	Free-ranging<br>Free-ranging	Shaving	Follicles absent	Fourie, Jolly *et al*., 2015
Grivet monkey (*Chlorocebus aethiops*)	Free-ranging	Cutting<br>Plucking	Follicles absent<br>Follicles present but manually removed before processing	Fourie, Turner *et al*., 2015
American black bear (*Ursus americanus*)	Free-ranging	Snagging	Follicles present, reference to D'Anna-Hernandez *et al.* 2011 (human hair cortisol concentration analysis)	Lafferty *et al*., 2015
Rhesus macaque (*Macaca mulatta*)	Captive	Shaving	Follicles absent	Qin, Chu *et al*., 2015
Rhesus macaque (*Macaca mulatta*)	Captive	Shaving	Follicles absent	Qin, Rizak *et al*., 2015
Eurasian badger (*Meles meles*)	Free-ranging	Snagging and collecting naturally shed hair	Hair shafts (no follicles) used for extraction	Agnew *et al*., 2016

Chimpanzee (*Pan troglodytes*)	Free-ranging	Collecting naturally shed hair	Follicles present but removed manually before processing	Carlitz *et al*., 2016
Red deer (*Cervus elaphus*)	Free-ranging	Stripping	Follicles present, full hair strands used for extraction	Caslini *et al*., 2016
Rhesus macaque (*Macaca mulatta*)	Captive	Shaving	Follicles absent	Feng *et al*., 2016
Yellow baboon (*Papio cynocephalus*)	Free-ranging	No details	No details, reference to Davenport *et al.* 2006	Fourie *et al*., 2016
Chacma baboon (*Papio ursinus*)	Free-ranging			
Guinea baboon (*Papio papio*)	Captive			
Allen's swamp monkey (*Allenopithecus nigroviridis*)	Captive			
Golden-bellied mangabey (*Cercocebus chrysogaster*)	Captive			
Gelada baboon (*Theropithecus gelada*)	Captive			
Western lowland gorilla (*Gorilla gorilla*)	Captive			
Red-cheeked gibbon (*Hylobates gabriellae*)	Captive			
Siamang (*Symphalangus syndactylus*)	Captive			
White-faced saki (*Pithecia pithecia*)	Captive			

Rhesus macaque (*Macaca mulatta*)	Captive	Shaving	Follicles absent	Lutz *et al*., 2016
Polar bear (*Ursus maritimus*)	Free-ranging	Shaving	Follicles absent	Mislan *et al*., 2016
Western fat-tailed dwarf lemur (*Cheirogaleus medius*)<br><br>Grey mouse lemur (*Microcebus murinus*)	Free-ranging<br><br>Free-ranging	Shaving	Follicles absent	Rakotoniaina *et al*., 2016
Dorcas gazelle (*Gazella dorcas*)	Captive	Shaving<br>Cutting	Follicles absent<br>Follicles absent	Salas *et al*., 2016
Rhesus macaque (*Macaca mulatta*)	Captive	Shaving	Follicles absent	Wooddell *et al*., 2016
Chimpanzee (*Pan troglodytes*)	Captive	Cutting	Follicles absent	Yamanashi, Teramoto, Morimura, Hirata, Inoue-Murayama *et al*., 2016
Chimpanzee (*Pan troglodytes*)	Captive	Cutting	Follicles absent	Yamanashi, Teramoto, Morimura, Hirata, Suzuki *et al*., 2016
Rhesus macaque (*Macaca mulatta*)	Captive	Shaving	Follicles absent	Zhang *et al*., 2016
Brown bear (*Ursus arctos*)	Captive	Shaving and plucking	Follicles absent and present in paired samples	Cattet *et al*., 2017
European hare	Captive	Shaving	Follicles absent	Esposito *et al*.,

(*Lepus europaeus*)				2017
Ring-tailed lemur (*Lemur catta*)	Free-ranging	No details	Entire hair shaft used for extraction, reference to Fourie *et al.* 2015	Fardi *et al*., 2017
Pig-tailed macaque (*Macaca nemestrina*)	Captive	Shaving	Follicles absent	Grant *et al*., 2017
Rhesus macaque (*Macaca mulatta*)	Captive	No details	No details, reference to Meyer *et al.* 2014	Hamel *et al*., 2017
Brown bear (*Ursus arctos*)<br><br>Polar bear (*Ursus maritimus*)	Free-ranging<br><br>Free-ranging	No details	No details, reference to Macbeth *et al.* 2010	Kroshko *et al*., 2017
Eastern chipmunk (*Tamias striatus*)	Free-ranging	Shaving	Follicles absent	Lyons *et al*., 2017
Polar bear (*Ursus maritimus*)	Free-ranging	No details	No details, cortisol assay protocol modified after Davenport *et al.* 2006 and Macbeth *et al.* 2012	Neuman-Lee *et al*., 2017
Rhesus macaque (*Macaca mulatta*)	Captive	Cutting	Follicles absent	Novak *et al*., 2017
Grey mouse lemur (*Microcebus murinus*)	Free-ranging	Shaving	Follicles absent	Rakotoniaina *et al*., 2017
Coyote (*Canis latrans*)	Captive	Shaving	Follicles absent	Schell *et al*., 2017
Brown bear (*Ursus arctos*)	Free-ranging	Plucking	Follicles present but removed manually before processing	Sergiel *et al*., 2017

		Cutting	Follicles absent	
Ring-tailed lemur (*Lemur catta*)	Captive	Shaving	Follicles absent	Tennenhouse *et al*., 2017
Rhesus macaque (*Macaca mulatta*)	Captive	Shaving	Follicles absent	Wooddell *et al*., 2017
Brown bear (*Ursus arctos*)	Free-ranging	Shaving<br>Plucking	Follicles absent<br>Follicles present but removed manually before processing	Cattet *et al*., 2018
Rhesus macaque (*Macaca mulatta*)	Captive	No details	No details, reference to Dettmer *et al.* 2015	Dettmer *et al*., 2018
Thinhorn sheep (*Ovis dalli dalli*)	Free-ranging	No details	No details; cortisol assay referenced to Davenport *et al.* 2006 and Koren *et al.* 2002	Downs *et al*., 2018
Rhesus macaques (*Macaca mulatta*)	Captive	Shaving	Follicles absent	Linden *et al.*, 2018
Common marmoset (*Callithrix jacchus*)<br>Tufted capuchin (*Cebus appella*)	Captive<br>Captive	Shaving<br>Shaving	Follicles absent	Phillips *et al*., 2018
Père David deer (*Elaphurus davidianus*)	Captive	Shaving	Follicles absent	Ping *et al.*, 2018
Alpine ibex (*Capra ibex ibex*)	Free-ranging	Shaving	Follicles absent	Prandi *et al.*, 2018
Roe deer (*Capreolus capreolus*)	Free-ranging	No details	No details; cortisol assay referenced to	Ventrella *et al.*, 2018

			Bacci *et al.* 2014	
Chimpanzee (*Pan troglodytes*)	Captive	Cutting	Follicles absent	Yamanashi *et al.*, 2018

List of references selected for the review on hair collection methods and types of samples used in studies that include hair cortisol concentrations measurement in wild mammal species

Agnew RCN, Smith VJ, Fowkes RC (2016) Wind turbines cause chronic stress in badgers (*Meles meles*) in Great Britain. *J Wildl Dis* 52: 459–467.

Ashley NT, Barboza PS, Macbeth BJ, Janz DM, Cattet MRL, Booth RK, Wasser SK (2011) Glucocorticosteroid concentrations in feces and hair of captive caribou and reindeer following adrenocorticotropic hormone challenge. *Gen Comp Endocrinol* 172: 382–391.

Bechshoft T, Derocher AE, Richardson E, Mislan P, Lunn NJ, Sonne C, Dietz R, Janz DM, St. Louis VL (2015) Mercury and cortisol in Western Hudson Bay polar bear hair. *Ecotoxicology* 24: 1315–1321.

Bechshøft T, Rigét FF, Sonne C, Letcher RJ, Muir DCG, Novak MA, Henchey E, Meyer JS, Eulaers I, Jaspers VLB, *et al.* (2012) Measuring environmental stress in East Greenland polar bears, 1892-1927 and 1988-2009: What does hair cortisol tell us? *Environ Int* 45: 15–21.

Bechshøft T, Sonne C, Dietz R, Born EW, Novak MA, Henchey E, Meyer JS (2011) Cortisol levels in hair of East Greenland polar bears. *Sci Total Environ* 409: 831–834.

Bechshøft T, Sonne C, Rigét FF, Letcher RJ, Novak MA, Henchey E, Meyer JS, Eulaers I, Jaspers VLB, Covaci A, *et al.* (2013) Polar bear stress hormone cortisol fluctuates with the North Atlantic Oscillation climate index. *Polar Biol* 36: 1525–1529.

Bechshøft, Sonne C, Dietz R, Born EW, Muir DCG, Letcher RJ, Novak MA, Henchey E, Meyer JS, Jenssen BM, *et al.* (2012) Associations between complex OHC mixtures and thyroid and cortisol hormone levels in East Greenland polar bears. *Environ Res* 116: 26–35.

Bourbonnais ML, Nelson TA, Cattet MRL, Darimont CT, Stenhouse GB (2013) Spatial analysis of factors influencing long-term stress in the grizzly bear (*Ursus arctos*) population of alberta, Canada. *PLoS One* 8(12): e83768.

Brearley G, McAlpine C, Bell S, Bradley A (2012) Influence of urban edges on stress in an arboreal mammal: A case study of squirrel gliders in southeast Queensland, Australia. *Landsc Ecol* 27: 1407–1419.

Bryan HM, Darimont CT, Paquet PC, Wynne-Edwards KE, Smits JEG (2013) Stress and reproductive hormones in grizzly bears reflect nutritional benefits and social consequences of a salmon foraging niche. *PLoS One* 8(11): e80537.

Bryan HM, Smits JEG, Koren L, Paquet PC, Wynne-Edwards KE, Musiani M (2015) Heavily hunted wolves have higher stress and reproductive steroids than wolves with lower hunting pressure. *Funct Ecol* 29: 347–356.

Carlitz EHD, Kirschbaum C, Miller R, Rukundo J, van Schaik CP (2015) Effects of body region and time on hair cortisol concentrations in chimpanzees (*Pan troglodytes*). *Gen Comp Endocrinol* 223: 9–15.

Carlitz EHD, Kirschbaum C, Stalder T, van Schaik CP (2014) Hair as a long-term retrospective cortisol calendar in orang-utans (*Pongo* spp.): New perspectives for stress monitoring in captive management and conservation. *Gen Comp Endocrinol* 195: 151–156.

Carlitz EHD, Miller R, Kirschbaum C, Gao W, Hänni DC, van Schaik CP (2016) Measuring hair cortisol concentrations to assess the effect of anthropogenic impacts on wild chimpanzees (*Pan troglodytes*). *PLoS One* 11(4): e0151870.

Caslini C, Comin A, Peric T, Prandi A, Pedrotti L, Mattiello S (2016) Use of hair cortisol analysis for comparing population status in wild red deer (*Cervus elaphus*) living in areas with different characteristics. *Eur J Wildl Res* 62: 713–723.

Cattet M, Macbeth BJ, Janz DM, Zedrosser A, Swenson JE, Dumond M, Stenhouse GB (2014) Quantifying long-term stress in brown bears with the hair cortisol concentration: A biomarker that may be confounded by rapid changes in response to capture and handling. *Conserv Physiol* 2: cou026.

Cattet M, Stenhouse GB, Boulanger J, Janz DM, Kapronczai L, Swenson JE, Zedrosser A (2018) Can concentrations of steroid hormones in brown bear hair reveal age class? *Conserv Physiol* 6: coy001.

Cattet M, Stenhouse GB, Janz DM, Kapronczai L, Anne Erlenbach J, Jansen HT, Nelson OL, Robbins CT, Boulanger J (2017) The quantification of reproductive hormones in the hair of captive adult brown bears and their application as indicators of sex and reproductive state. *Conserv Physiol* 5: cox032.

Chu X, Rizak JD, Yang S, Wang J, Ma Y, Hu X (2014) A natural model of behavioral depression in postpartum adult female cynomolgus monkeys (Macaca fascicularis). *Zool Res* 35: 174–181.

Clara E, Tommasi L, Rogers LJ (2008) Social mobbing calls in common marmosets (*Callithrix jacchus*): Effects of experience and associated cortisol levels. *Anim Cogn* 11: 349–358.

Davenport MD, Lutz CK, Tiefenbacher S, Novak MA, Meyer JS (2008) A rhesus monkey model of self-injury: Effects of relocation stress on behavior and neuroendocrine function. *Biol Psychiatry* 63: 990–996.

Davenport MD, Tiefenbacher S, Lutz CK, Novak MA, Meyer JS, Kirschbaum C, Tietze A, Skoluda N, Dettenborn L, Raul JS, *et al.* (2006) Analysis of endogenous cortisol concentrations in the hair of rhesus macaques. *Psychoneuroendocrinology* 37: 1105–1111.

Dettmer AM, Murphy AM, Guitarra D, Slonecker E, Suomi SJ, Rosenberg KL, Novak MA, Meyer JS, Hinde K (2018) Cortisol in neonatal mother's milk predicts later infant social and cognitive functioning in rhesus monkeys. *Child Dev* 89: 525–538.

Dettmer AM, Novak MA, Meyer JS, Suomi SJ (2014) Population density-dependent hair cortisol concentrations in rhesus monkeys (*Macaca mulatta*). *Psychoneuroendocrinology* 42: 59-67.

Dettmer AM, Novak MA, Suomi SJ, Meyer JS (2012) Physiological and behavioral adaptation to relocation stress in differentially reared rhesus monkeys: Hair cortisol as a biomarker for anxiety-related responses. *Psychoneuroendocrinology* 37: 191–199.

Dettmer AM, Novak MFSX, Novak MA, Meyer JS, Suomi SJ (2009) Hair cortisol predicts object permanence performance in infant rhesus macaques (*Macaca mulatta*). *Dev Psychobiol* 51: 706–713.

Dettmer AM, Rosenberg K, Menard MT, Suomi SJ, Meyer JS (2015) Differential maternal investment in rhesus monkey mothers with hair loss in the neonatal period. *Am J Phys Anthropol* 156: 119–120.

Dettmer AM, Rosenberg KL, Suomi SJ, Meyer JS, Novak MA, Chavatte-Palmer P (2015) Associations between parity, hair hormone profiles during pregnancy and lactation, and infant development in rhesus monkeys (*Macaca mulatta*). *PLoS One* 10(7): e0131692.

Downs CJ, Boan B V., Lohuis TD, Stewart KM (2018) Investigating relationships between reproduction, immune defenses, and cortisol in Dall sheep. *Front Immunol* 9: 105.

Esposito L, Auletta L, Ciani F, Pelagalli A, Pasolini MP, Lamagna B, Piscopo N, Amici A (2017) Hair cortisol levels in captive brown hare (*Lepus europaeus*): potential effect of sex, age, and breeding technology. *Eur J Wildl Res* 63: 62.

Fairbanks LA, Jorgensen MJ, Bailey JN, Breidenthal SE, Grzywa R, Laudenslager ML (2011) Heritability and genetic correlation of hair cortisol in vervet monkeys in low and higher stress environments. *Psychoneuroendocrinology* 36: 1201–1208.

Fardi S, Sauther ML, Cuozzo FP, Jacky IAY, Bernstein RM (2017) The effect of extreme weather events on hair cortisol and body weight in a wild ring-tailed lemur population (*Lemur catta*) in southwestern Madagascar. *Am J Primatol* 80: e22731.

Feng X, Wu X, Morrill RJ, Li Z, Li C, Yang S, Li Z, Cui D, Lv L, Hu Z, *et al.* (2016) Social correlates of the dominance rank and long-term cortisol levels in adolescent and adult male rhesus macaques (*Macaca mulatta*). *Sci Rep* 6: 25431.

Fourie NH, Bernstein RM (2011) Hair cortisol levels track phylogenetic and age related differences in hypothalamic-pituitary-adrenal (HPA) axis activity in non-human primates. *Gen Comp Endocrinol* 174: 150–155.

Fourie NH, Brown JL, Jolly CJ, Phillips-Conroy JE, Rogers J, Bernstein RM (2016) Sources of variation in hair cortisol in wild and captive non-human primates. *Zoology* 119: 119–125.

Fourie NH, Jolly CJ, Phillips-Conroy JE, Brown JL, Bernstein RM (2015) Variation of hair cortisol concentrations among wild populations of two baboon species (*Papio anubis*, *P. hamadryas*) and a population of their natural hybrids. *Primates* 56: 259–272.

Fourie NH, Turner TR, Brown JL, Pampush JD, Lorenz JG, Bernstein RM (2015) Variation in vervet (*Chlorocebus aethiops*) hair cortisol concentrations reflects ecological disturbance by humans. *Primates* 56: 365–373.

Grant K, Worlein J, Meyer J, Novak M, Kroeker R, Rosenberg K, Kenney C BT (2018) A Longitudinal study of hair cortisol concentrations in *Macaca nemestrina* mothers and infants. *Am J Primatol* 79(2): 1–9.

Hamel AF, Lutz CK, Coleman K, Worlein JM, Peterson EJ, Rosenberg KL, Novak MA MJ (2017) Responses to the human intruder test are related to hair cortisol phenotype and sex in rhesus macaques (*Macaca mulatta*). *Am J Primatol* 79(1): 1–10.

Kapoor A, Lubach G, Hedman C, Ziegler TE, Coe CL (2014) Hormones in infant rhesus monkeys' (*Macaca mulatta*) hair at birth provide a window into the fetal environment. *Pediatr Res* 75: 476–481.

Koren L, Mokady O, Karaskov T, Klein J, Koren G, Geffen E (2002) A novel method using hair for determining hormonal levels in wildlife. *Anim Behav* 63: 403–406.

Kroshko T, Kapronczai L, Cattet MRL, Macbeth BJ, Stenhouse GB, Obbard ME, Janz DM (2017) Comparison of methanol and isopropanol as wash solvents for determination of hair cortisol concentration in grizzly bears and polar bears. *MethodsX* 4: 68–75.

Lafferty DJR, Laudenslager ML, Mowat G, Heard D, Belant JL (2015) Sex, diet, and the social environment: Factors influencing hair cortisol concentration in free-ranging black bears (*Ursus americanus*). *PLoS One* 10(11): e0141489.

Laudenslager ML, Natvig C, Corcoran CA, Blevins MW, Pierre PJ, Bennett AJ (2013) The influences of perinatal challenge persist into the adolescent period in socially housed bonnet macaques (*Macaca radiata*). *Dev Psychobiol* 55: 316–322.

Linden JB, Capitanio JP, McCowan B, Isbell LA (2018) Coping style and cortisol levels in infancy predict hair cortisol following new group formation in captive rhesus macaques (Macaca mulatta). *Am J Primatol* 80(12): 1–13.

Lutz CK, Coleman K, Worlein JM, Kroeker R, Menard MT, Rosenberg K, Meyer JS, Novak MA (2016) Factors influencing alopecia and hair cortisol in rhesus macaques (*Macaca mulatta*). *J Med Primatol* 45: 180–188.

Lyons J, Mastromonaco G, Edwards DB, Schulte-Hostedde AI (2017) Fat and happy in the city: Eastern chipmunks in urban environments. *Behav Ecol* 28: 1464–1471.

Macbeth BJ, Cattet MRL, Obbard ME, Middel K, Janz DM (2012) Evaluation of hair cortisol concentration as a biomarker of long-term stress in free-ranging polar bears. *Wildl Soc Bull* 36: 747–758.

Macbeth BJ, Cattet MRL, Stenhouse GB, Gibeau ML, Janz DM (2010) Hair cortisol concentration as a noninvasive measure of long-term stress in free-ranging grizzly bears (*Ursus arctos*): considerations with implications for other wildlife. *Can J Zool* 88: 935–949.

Malcolm KD, McShea WJ, Van Deelen TR, Bacon HJ, Liu F, Putman S, Zhu X, Brown JL (2013) Analyses of fecal and hair glucocorticoids to evaluate short- and long-term stress and recovery of Asiatic black bears (*Ursus thibetanus*) removed from bile farms in China. *Gen Comp Endocrinol* 185: 97–106.

Mastromonaco GF, Gunn K, McCurdy-Adams H, Edwards DB, Schulte-Hostedde AI (2014) Validation and use of hair cortisol as a measure of chronic stress in eastern chipmunks (*Tamias striatus*). *Conserv Physiol* 2: cou055.

Meyer J, Novak M, Hamel A, Rosenberg K (2014) Extraction and analysis of cortisol from human and monkey hair. *J Vis Exp* 83: e50882.

Mislan P, Derocher AE, St. Louis VL, Richardson E, Lunn NJ, Janz DM (2016) Assessing stress in Western Hudson Bay polar bears using hair cortisol concentration as a biomarker. *Ecol Indic* 71: 47–54.

Neuman-Lee LA, Terletzky PA, Atwood TC, Gese EM, Smith GD, Greenfield S, Pettit J, French SS (2017) Demographic and temporal variations in immunity and condition of polar bears (*Ursus maritimus*) from the southern Beaufort Sea. *J Exp Zool Part A Ecol Integr Physiol* 327: 333–346.

Novak MA, Hamel AF, Coleman K, Lutz CK, Worlein J, Menard M, Ryan A, Rosenberg K, Meyer JS (2014) Hair loss and hypothalamic-pituitary-adrenocortical axis activity in captive rhesus macaques (*Macaca mulatta*). *J Am Assoc Lab Anim Sci* 53: 261–266.

Novak MA, Menard MT, El-Mallah SN, Rosenberg K, Corrine K, Worlein J, Coleman K, Meyer JS (2017) Assessing significant (> 30%) alopecia as a possible biomarker for stress in captive rhesus monkeys (*Macaca mulatta*). *Am J Primatol* 79(1): 1-8.

Phillips KA, Tukan AN, Rigodanzo AD, Reusch RT, Brasky KM, Meyer JS (2018) Quantification of hair cortisol concentration in common marmosets (*Callithrix jacchus*) and tufted capuchins (*Cebus apella*). *Am J Primatol* 80(7): e22879.

Ping X, Liu N, Jiang Z, Xu H, Bai J, Zhang S, Li C (2018) Assaying progesterone, estradiol and cortisol concentrations in hair of Père David deer hinds: an alternative way to reflect seasonality of steroid secretion. *Biol Rhythm Res* 49(3): 422–430.

Prandi A, Peric T, Corazzin M, Comin A, Colitti M (2018) A first survey on hair cortisol of an alpine ibex (Capra ibex ibex) population. *Anim Sci Pap Rep* 36(1): 57–74.

Qin D, Dominic Rizak J, Feng X, Chu X, Yang S, Li C, Lv L, Ma Y, Hu X (2013) Social rank and cortisol among female rhesus macaques (*Macaca mulatta*). *Zool Res* 34: E42–E49.

Qin D, Chu X, Feng X, Li Z, Yang S, Lü L, Yang Q, Pan L, Yin Y, Li J, *et al.* (2015) The first observation of seasonal affective disorder symptoms in Rhesus macaque. *Behav Brain Res* 292: 463–469.

Qin D, Rizak J, Feng X, Yang S, Yang L, Fan X, Lü L, Chen L, Hu X (2015) Cortisol responses to chronic stress in adult macaques: Moderation by a polymorphism in the serotonin transporter gene. *Behav Brain Res* 278: 280–285.

Rakotoniaina JH, Kappeler PM, Kaesler E, Hämäläinen AM, Kirschbaum C, Kraus C (2017) Hair cortisol concentrations correlate negatively with survival in a wild primate population. *BMC Ecol* 17: 30.

Rakotoniaina JH, Kappeler PM, Ravoniarimbinina P, Pechouskova E, Hämäläinen AM, Grass J, Kirschbaum C, Kraus C (2016) Does habitat disturbance affect stress, body condition and parasitism in two sympatric lemurs? *Conserv Physiol* 4: cow034.

Salas M, Temple D, Abáigar T, Cuadrado M, Delclaux M, Enseñat C, Almagro V, Martínez-Nevado E, Quevedo MÁ, Carbajal A, *et al.* (2016) Aggressive behavior and hair cortisol levels in captive Dorcas gazelles (*Gazella dorcas)* as animal-based welfare indicators. *Zoo Biol* 35: 467–473.

Schell CJ, Young JK, Lonsdorf E V., Mateo JM, Santymire RM (2017) Investigation of techniques to measure cortisol and testosterone concentrations in coyote hair. *Zoo Biol* 36: 220–225.

Sergiel A, Hobson KA, Janz DM, Cattet M, Selva N, Kapronczai L, Gryba C, Zedrosser A (2017) Compatibility of preparatory procedures for the analysis of cortisol concentrations and stable isotope (δ13C, δ15N) ratios: a test on brown bear hair. *Conserv Physiol* 5: cox021.

Tennenhouse EM, Putman S, Boisseau NP, Brown JL (2017) Relationships between steroid hormones in hair and social behaviour in ring-tailed lemurs (*Lemur catta*). *Primates* 58: 199–209.

Terwissen C V., Mastromonaco GF, Murray DL (2013) Influence of adrenocorticotrophin hormone challenge and external factors (age, sex, and body region) on hair cortisol concentration in Canada lynx (*Lynx canadensis*). *Gen Comp Endocrinol* 194: 162–167.

Ventrella D, Elmi A, Barone F, Carnevali G, Govoni N, Bacci ML (2018) Hair testosterone and cortisol concentrations in pre- and post-rut roe deer bucks: Correlations with blood levels and testicular morphometric parameters. *Animals* 8(7): 113.

Wooddell LJ, Hamel AF, Murphy AM, Byers KL, Kaburu SSK, Meyer JS, Suomi SJ, Dettmer AM (2017) Relationships between affiliative social behavior and hair cortisol concentrations in semi-free ranging rhesus monkeys. *Psychoneuroendocrinology* 84: 109–115.

Wooddell LJ, Kaburu SSK, Rosenberg KL, Meyer JS, Suomi SJ, Dettmer AM (2016) Matrilineal behavioral and physiological changes following the removal of a non-alpha matriarch in rhesus macaques (*Macaca mulatta*). *PLoS One* 11(6): e0157108.

Yamanashi Y, Morimura N, Mori Y, Hayashi M, Suzuki J (2013) Cortisol analysis of hair of captive chimpanzees (*Pan troglodytes*). *Gen Comp Endocrinol* 194: 55–63.

Yamanashi Y, Teramoto M, Morimura N, Hirata S, Inoue-Murayama M, Idani G (2016) Effects of relocation and individual and environmental factors on the long-term stress levels in captive chimpanzees (*Pan troglodytes*): monitoring hair cortisol and behaviors. *PLoS One* 11(7): e0160029.

Yamanashi Y, Teramoto M, Morimura N, Hirata S, Suzuki J, Hayashi M, Kinoshita K, Murayama M, Idani G (2016) Analysis of hair cortisol levels in captive chimpanzees: Effect of various methods on cortisol stability and variability. *MethodsX* 3: 110–117.

Yamanashi Y, Teramoto M, Morimura N, Nogami E, Hirata S (2018) Social relationship and hair cortisol level in captive male chimpanzees (*Pan troglodytes*). *Primates* 59: 145–152.

Zhang Z, Mao Y, Feng X, Zheng N, Lü L, Ma Y, Qin D, Hu X (2016) Early adversity contributes to chronic stress induced depression-like behavior in adolescent male rhesus monkeys. *Behav Brain Res* 306: 154–159.
